# Entwicklung und Validierung einer Checkliste zur Bewertung von Videos zum Erlernen von Reanimationsmaßnahmen

**DOI:** 10.1007/s00063-021-00857-6

**Published:** 2021-09-01

**Authors:** J. Sterz, P. R. Tückmantel, L. Bepler, M. C. Stefanescu, Y. Gramlich, A. Flinspach, M. Rüsseler

**Affiliations:** 1 Klinik für Unfall‑, Hand- und Wiederherstellungschirurgie, Universitätsklinikum Frankfurt, Goethe-Universität, Theodor-Stern-Kai 7, 60590 Frankfurt, Deutschland; 2 Frankfurter Interdisziplinäres Simulationstraining, Fachbereich 16, Goethe-Universität, Frankfurt am Main, Deutschland; 3 Abteilung für Unfallchirurgie und Orthopädische Chirurgie, BG Unfallklinik Frankfurt am Main, Frankfurt am Main, Deutschland; 4 Klinik für Anästhesiologie, Intensivmedizin und Schmerztherapie, Universitätsklinikum Frankfurt, Goethe-Universität, Frankfurt, Deutschland

**Keywords:** Lehrvideos, Qualitätssicherung, Reanimation, Checkliste, Lehrforschung, Instructional videos, Quality assurance, Resuscitation, Checklist, Educational research

## Abstract

**Hintergrund:**

Gut durchgeführte Wiederbelebungsmaßnahmen können bei einem Herz-Kreislauf-Stillstand das Outcome verbessern. Um praktische Fähigkeiten zu erlernen, greifen Medizinstudierende oft auf Lehrvideos zurück. Studien zeigen jedoch häufig eine unzureichende Qualität der im Internet zur Verfügung gestellten Videos zu Reanimationsmaßnahmen. Eine Bewertung anhand einer validierten, auf den aktuellen „guidelines“ basierten Checkliste fehlt bisher.

**Ziel der Arbeit:**

Entwicklung und Validierung einer Checkliste zur Bewertung von Lehrvideos zur Reanimation.

**Material und Methoden:**

In einem Expertenworkshop erfolgte basierend auf den aktuellen „guidelines“ die Formulierung der Checklistenitems. Die Checkliste wurde in einem vierstufigen Reviewprozess von Notärzten getestet. Die Bewertungen wurden analysiert und die Items angepasst und spezifiziert.

Nach dem Reviewprozess wurde die Checkliste an 74 Videos zur Reanimation angewendet.

**Ergebnisse:**

Die Checkliste umfasst 25 Items in vier Kategorien (initiale Maßnahmen, Thoraxkompression, AED-Nutzung, Atmung), die auf einer 3‑stufigen Likert-Skala bewertet werden. 16 NotärztInnen nahmen an der Studie teil. Sie bewerteten jeweils durchschnittlich 9,3 ± 5,7 Videos. Die Reviewer stimmten in 65,1 ± 12,6 % der Fälle überein. Die höchsten Übereinstimmungen wurden im Unterthema AED erzielt, das Item „Beim Schock Patienten nicht berühren“ wies die höchste Übereinstimmung auf. Die Items der Kategorie Thoraxkompression wurden am häufigsten unterschiedlich bewertet.

**Diskussion:**

Es konnte erstmalig für den deutschsprachigen Raum eine Checkliste zur Bewertung von Lehrvideos zur Reanimation erstellt und validiert werden.

## Abstract (en)

**Background:**

Well-performed resuscitation measures can improve the outcome in the event of cardiovascular arrest. Medical students often use teaching videos to learn practical skills. Studies confirmed the often inadequate quality of the videos on resuscitation available on the Internet. An evaluation using a validated checklist based on the current guidelines has so far been lacking.

**Objective:**

The development and validation of a checklist for evaluating instructional videos on resuscitation.

**Material and methods:**

In an expert workshop, checklist items were formulated based on the current guidelines. The checklist was tested by emergency physicians in a 4-step review process. The evaluations were analyzed and the items adjusted and specified if necessary.

After the review process was completed, the checklist was applied to 74 videos on the topic of resuscitation.

**Results:**

The checklist consists of 25 items in 4 categories (initial measures, chest compression, AED use, breathing), which are rated on a 3-level Likert scale.

A total of 16 emergency doctors participated in the study and rated an average of 9.3 ± 5.7 videos each. The reviewers agreed in 65.1 ± 12.6% of the cases. The highest agreement was achieved in the subtopic AED, with the item “do not touch patients in shock” having the highest agreement. The items in the thoracic compression category were most often rated differently.

**Conclusion:**

For the first time, a checklist for evaluating instructional videos for resuscitation was created and validated for German-speaking countries.

Obwohl außerklinische Reanimationen eine hohe Inzidenz aufweisen, bestehen bei Medizinstudierenden häufig Kompetenzdefizite. Obwohl Lehrvideos gerade für unplanbare, aufgrund ethischer Problematiken nicht am Patientenbett unterrichtbare Fertigkeiten wie die Reanimation ein gut untersuchtes Lehrmedium sind, fehlen besonders bei frei zugänglichen Videoplattformen wie YouTube Qualitätskontrollen. In der vorliegenden Arbeit wurde daher eine Checkliste zur Bewertung von Lehrvideos zur Reanimation entwickelt und validiert.

## Hintergrund und Fragestellung

Die Inzidenz außerklinischer Reanimationen liegt in Deutschland bei 116/100.000 pro Jahr [22]. Obwohl sich in über 90 % der Notfälle Ersthelfer vor Ort befinden [3], werden in Deutschland nur 43,5 % der Patienten mit Herzstillstand durch anwesende Laien reanimiert [22], insgesamt liegt Deutschland im europäischen Vergleich bezüglich der Anzahl der Laienreanimationen im unteren Drittel [23]. Als Gründe hierfür wurden Panik (37,5 %), Angst vor falscher Durchführung (10,8 %) und Angst, die Person zu verletzen (1,8 %), angegeben [20].

Jedoch zeigen sich nicht nur bei medizinischen Laien, sondern bereits bei Medizinstudierenden deutliche Kompetenzdefizite: Baldi et al. konnten zeigen, dass selbst bei Medizinstudierenden deutliche Kompetenzdefizite bestehen: Nur 57,8 % der europäischen Medizinstudierenden kennen die korrekte Kompressionsrate [2]. Zudem sind vielen Mitarbeitern im Gesundheitswesen Aspekte wie die Drucktiefe oder der korrekte Druckpunkt unklar [4, 16].

Aufgrund der fast ubiquitären Nutzbarkeit bieten online verfügbare Videos ein häufig genutztes Lernmedium. Medizinstudierende geben an, mit Videos effektiver lernen zu können als mit Büchern bzw. reinen Texten oder Bildern [6]. Besonders bei praktischen Fertigkeiten greifen sie oft auf Lehrvideos zurück [11, 24]. Die Videoplattform YouTube kann hierbei eine hilfreiche Unterstützung sein [21]. Neben hochwertigen Videos in vielen YouTube-Kanälen gibt es sehr viele Videos mit falschen oder veralteten Informationen [5, 15]. Da es für die meisten Inhalte keine Qualitätskontrolle gibt, ist die Gefahr groß, dass durch das Lernen mit diesen Videos Fehler und falsche Abläufe erlernt werden.

Yaylaci et al. analysierten 2014 die Qualität von YouTube-Videos zur Reanimation, dabei zeigte sich, dass nur 11,5 % der Videos die notwendigen Maßnahmen in der korrekten Reihenfolge darstellten. Die Reliabilität der erstellten Checkliste bestehend aus sieben Items wurde nicht untersucht [25]. Weitere Studien zu dem Thema bestätigten die häufig unzureichende Qualität der im Internet zur Verfügung gestellten Videos zur Reanimation [10, 14]. In keiner dieser Arbeiten erfolgte jedoch die Verwendung einer validierten, auf den aktuellen „guidelines“ [1] zur kardiopulmonalen Reanimation basierten Checkliste.

Zwar existiert zur themenunabhängigen Bewertung von Lehrvideos hinsichtlich der didaktischen Ausgestaltung bereits eine validierte Checkliste [17], für die kardiopulmonale Reanimation fehlt aber eine darüber hinausgehende inhaltliche Bewertungscheckliste. Ziel der vorliegenden Arbeit war daher die Entwicklung und Implementierung einer Checkliste zur inhaltlichen Bewertung von Videos zur Reanimation mit dem langfristigen Ziel, Lehrenden und Lernenden qualitativ überprüfte, frei zugängliche Videos zur Verfügung zu stellen.

## Studiendesign und Untersuchungsmethoden

### Erarbeitung der Checklistenitems

In einem Expertenworkshop wurden basierend auf den aktuellen „guidelines“ die im Rahmen der präklinischen Reanimation durchzuführenden Schritte identifiziert und vier Kategorien zugeordnet: initiale Maßnahmen, Thoraxkompression, Verwendung des AED und Atmung. Mitglieder des Expertenworkshops waren neben medizindidaktischen (Absolventen des Masterstudiengangs Medical Education) und fachlichen Experten (Notärzt:innen mit langjähriger Berufserfahrung) auch Medizinstudierende als Vertreter der Zielgruppe der Lehrvideos.

Zu den identifizierten Schritten wurden kurze, präzise Checklistenitems formuliert. Diese Items wurden im Rahmen des Workshops an Beispielsvideos getestet, im Hinblick auf ihre Verständlichkeit und Eignung analysiert und, wenn nötig, angepasst oder spezifiziert. Die Bewertung erfolgte auf einer 3‑stufigen Skala (0 = nicht erwähnt; 1 = falsch oder unvollständig durchgeführt und 2 = richtig durchgeführt).

Optimierung und Analyse der Checkliste:

Die so erstellte erste Version der Checkliste wurde in einem vierstufigen Reviewprozess hinsichtlich Qualität, Verständlichkeit und Anwendbarkeit getestet und überarbeitet. Hierfür bewerteten jeweils zwei bis drei nicht an der Erstellung der Checkliste beteiligte NotärztInnen jeweils zwei Videos. Die Bewertung wurde ohne vorherige Schulung und ohne die Möglichkeit zu Absprachen zwischen den Reviewern durchgeführt. Analysiert wurden Videos der Plattform YouTube, die unter den folgenden Suchbegriffen gefunden werden konnten:

- Thoraxkompression
- Herzdruckmassage
- CPR
- Cardiac Massage
- Cardiopulmonary Resuscitation
- Basic Life Support
- Herzstillstand Erste Hilfe
- Herz Erste Hilfe
- Reanimation
- Wiederbelebung

Ausgeschlossen wurden Videos, die offensichtlich nicht ernst gemeint waren, keinen Ton oder kein Bild hatten, Videos in anderen Sprachen als Deutsch oder Englisch, die Reanimationen an Kindern oder Tieren zeigten oder die reale, intraoperative oder Reanimation unter Verwendung einer mechanischen Kompressionshilfe zeigten.

Im Anschluss wurden die einzelnen Bewertungen analysiert und von den Reviewern sowie den Autoren gemeinsam kritisch insbesondere im Hinblick auf Gründe für die jeweilige Punktevergabe, Stärken und Schwächen der Items diskutiert. Basierend hierauf wurden die Items angepasst und spezifiziert. An der folgenden Reviewrunde nahmen dann nur NotärztInnen teil, die bisher noch nicht beteiligt waren.

Dieses mehrstufige Verfahren diente der Qualitätssicherung und Validierung der Checkliste hinsichtlich der Testgütekriterien Objektivität, Reliabilität und Validität. Hierbei bezeichnet die Reliabilität den Grad der Genauigkeit, mit dem das geprüfte Merkmal gemessen wird, sowie die Wiederholbarkeit der Messung. Ein wichtiger Aspekt hierbei ist die Überprüfung, ob verschiedene ReviewerInnen dasselbe Video identisch beurteilen. Hierfür erfolgte die Analyse der Übereinstimmung zwischen den Reviewern.

Die Objektivität lässt sich in mehrere Unterformen unterteilen, unter anderem die Durchführungs- und die Auswertungsobjektivität. Durch sie soll gewährleistet werden, dass die Bewertung eines Videos unabhängig von den Reviewern ist. Hierfür ist besonders eine präzise Formulierung der Items von Bedeutung. Dies wurde in der vorliegenden Arbeit durch die wiederholte Diskussion der Interpretation der Items zwischen den ReviewerInnen sichergestellt.

Die Validität eines Messinstruments gibt an, wie gut das Instrument das misst, was es zu messen vorgibt. Hierbei wird die Inhaltsvalidität von der Kriteriumsvalidität unterschieden. Die Kriteriumsvalidität beschreibt den Grad der Übereinstimmung des Messinstruments mit einem Goldstandard. Da dieser für die Bewertung von Lehrvideos zur Reanimation bisher nicht vorliegt, wurde ein besonderes Augenmerk auf die Inhaltsvalidität gelegt. Diese beschreibt, ob und in welchem Maß die Checklistenitems den zu messenden Merkmalsbereich ausreichend genau repräsentieren. Dies wurde durch die Erstellung der Checklistenitems basierend auf den aktuellen „guidelines“, die Erstellung im Rahmen eines Expertenworkshops und die Anwendung im Rahmen des Reviewprozesses sichergestellt.

Die so erstellte Checkliste wurde an insgesamt 74 Videos zum Thema Reanimation, die basierend auf den oben beschriebenen Ein- und Ausschlusskriterien identifiziert werden konnten, angewendet. Hierbei wurde jedes Video von insgesamt 2 NotärztInnen bewertet. Als ReviewerInnen wurden gezielt NotärztInnen aus verschiedenen deutschen Städten und sowohl universitär arbeitende als auch nichtuniversitär arbeitende ÄrztInnen rekrutiert. Die Teilnahme erfolgte freiwillig nach mündlicher und schriftlicher Aufklärung.

### Datenauswertung

Die Datenerfassung sowie Auswertung der Mittelwerte, Standardabweichung, einzelnen Items beider Checklisten und der Vergleichbarkeit der beiden ReviewerInnen erfolgte in Microsoft Excel (Microsoft Corporation, Redmond, USA).

## Ergebnisse

Die resultierende Checkliste umfasst 25 Items in vier Kategorien. Sieben Items entfallen auf die Kategorie ‚initiale Maßnahmen‘, acht auf ‚Thoraxkompression‘, sechs auf ‚AED-Nutzung‘ und vier auf ‚Atmung‘. Die Bewertung der Items erfolgt auf einer 3‑stufigen Likert-Skala (0 = nicht erwähnt; 1 = falsch oder unvollständig durchgeführt und 2 = richtig durchgeführt). Während des Reviewverfahrens zeigte sich, dass nicht immer alle Items auf alle Videos zutreffend sind. Daher wurde die Option ergänzt, Items auszuschließen, falls das Item im Kontext des Videos nicht zutrifft oder bereits erfolgt war.

Die Items der ‚AED-Nutzung‘ und ‚Atmung‘ sind optional, d. h., falls die Nutzung des AED bzw. die Atmung nicht im Video behandelt wird, besteht die Möglichkeit, die Auswahl „nicht vorhanden“ bzw. „nicht zutreffend“ für die gesamte Untergruppe zu wählen. Grund hierfür ist, dass die Beatmung durch Laien ohne Training nicht mehr empfohlen wird. Zusätzlich fällt diese Untergruppe bei erfolgter Intubation weg. In diesem Fall werden die Items dieser Untergruppen aus der Gesamtwertung des Videos ausgeschlossen. Insgesamt ergibt sich so eine erreichbare Gesamtpunktzahl von mindestens 22 bis zu 50 maximalen Punkten.

Während des Reviewprozesses zeigte sich, dass zur eindeutigen Beantwortung der Checkliste eine kurze Anleitung nötig ist, die den Umgang mit der Option „nicht zutreffend/bereits erfolgt“ erklärt und klarstellt, dass Maßnahmen nur dann als richtig bewertet werden dürfen, wenn sie sowohl richtig durchgeführt als auch angesagt wurden. Hiervon ausgenommen sind nur die Items „Kompressor nach 2 min wechseln“ und „Zyklen wiederholen, bis Hilfe eintrifft“, bei denen das Erläutern der Maßnahmen ausreichend ist. Wird eine andere Maßnahme nur benannt, aber nicht gezeigt, ist die Bewertung „unvollständig/falsch durchgeführt“ zu wählen.

Die erstellte Checkliste zur inhaltlichen Bewertung von Lehrvideos zur Reanimation ist diesem Artikel als Supplement beigefügt.

## Übereinstimmung der beiden ReviewerInnen

### ReviewerInnen

Tab. 1 zeigt die Verteilung der NotärztInnen nach Geschlecht, Fachrichtung und Anzahl der bewerteten Videos. Die ReviewerInnen bewerteten im Durchschnitt 9,25 Videos ± 5,69.

**Table 1:** 

Reviewer	Fachrichtung	Geschlecht
1	Unfallchirurgie	M
2	Innere	M
3	Anästhesie	M
4	Mund‑, Kiefer‑, Gesichtschirurgie	M
5	Unfallchirurgie	W
6	Unfallchirurgie	W
7	Unfallchirurgie	W
8	Anästhesie	W
9	Anästhesie	W
10	Pädiatrie	M
11	Anästhesie	M
12	Unfallchirurgie	M
13	Unfallchirurgie	M
14	Unfallchirurgie	W
15	Unfallchirurgie	M
16	Unfallchirurgie	M

Insgesamt stimmten die ReviewerInnen in 65,06 ± 12,56 % der Fälle überein. Die höchsten Übereinstimmungen erzielten die ReviewerInnen im Unterthema AED, wobei das Item „Beim Schock Patienten nicht berühren“ mit 85,14 % die höchste Übereinstimmung aufwies. Die Items der Kategorie Thoraxkompression wurden am häufigsten unterschiedlich bewertet. Dabei liegt die Übereinstimmung für das Item „Unterbrechung < 10 Sek“ bei 47,29 %, die Items „Kompressionstiefe 5 cm“ und „Entlastung“ werden jeweils in 48,64 % der Fälle gleich bewertet. Tab. 2 zeigt die Übereinstimmung der ReviewerInnen für die jeweiligen Items der Checkliste.

**Table 2:** 

Item	Übereinstimmung [%]
*Initiale Maßnahmen*	
Ansprechen	64,86
Schmerzreiz	67,56
Atemwege	52,70
Atmung prüfen	63,51
Notruf	68,91
Entkleiden	48,64
Hinlegen	56,75
*Thoraxkompression*	
Harte Unterlage	54,05
100–120/Min	66,22
Korrekter Druckpunkt	58,11
Kompressionstiefe 5 cm	48,65
Entlastung	48,65
Kompressor tauschen	62,16
Zyklen wiederholen	52,70
Unterbrechung < 10 Sek	47,29
*AED*	
Nicht vorhanden → Unterpunkte fallen weg	91,89
2. Priorität	67,57
Elektroden aufkleben	75,68
Rhythmusanalyse	83,78
Beim Schock Pat nicht berühren	85,14
Sofort nach Schock Thoraxkompression	83,78
Erneute Rhythmuskontrolle nach 2 Min	81,08
*Atmung*	
Nicht zutreffend, da Laien-Reanimation → Unterpunkte fallen weg	77,03
30 zu 2	66,22
Kopf überstrecken	62,16
Nase zuhalten über den Mund beatmen/Maske mit C‑Griff	58,11
Auf Thoraxhebung als Feedback	63,51

## Diskussion

In der vorliegenden Arbeit wurde erstmals eine auf den aktuellen „guidelines“ [1] basierende Checkliste für die Qualität von Lehrvideos zum Thema Reanimation erstellt und validiert. Diese Checkliste bewertet die Videos anhand von 25 Items in vier Kategorien.

Obwohl die entwickelte Checkliste im Rahmen eines ausführlichen, mehrstufigen Reviewverfahrens mehrfach angepasst und validiert wurde, ist die Übereinstimmung zwischen den ReviewerInnen im Rahmen der Anwendung an insgesamt 74 Videos mit 65,06 ± 12,56 % moderat. Hierbei zeigt sich eine große Spanne von einer Übereinstimmung von 85,14 % für das Item „Beim Schock Patienten nicht berühren“ bis zu 47,29 % für das Item „Unterbrechung < 10 Sek“. Diese Diskrepanz zeigt, wie schwer es selbst für erfahrene NotärztInnen ist, die Qualität der durchgeführten Maßnahmen einzuschätzen, insbesondere wenn es sich um Kriterien handelt, die nicht mit dem bloßen Auge abzuschätzen sind (z. B. exakte Drucktiefe, konkrete Zeitspannen). Dies entspricht auch den bisher publizierten Analysen von Lehrvideos zur Behandlung von medizinischen Notfällen: Viele Arbeiten, die Videos anhand von Checklisten bewerteten, geben Übereinstimmung zwischen den ReviewerInnen nicht an [7, 12, 18]. So bewerten beispielsweise in den Arbeiten von Elicabuk et al. und Katipoğlu et al. zwei ReviewerInnen Lehrvideos zur Reanimation. Wichen die Bewertungen dieser beiden ReviewerInnen in einem Item voneinander ab, bewertete ein dritter Reviewer das Video und diejenige Bewertung wurde für das Gesamtergebnis berücksichtig, die die Mehrheit der ReviewerInnen ausgewählt hatte [7, 12].

Die Ergebnisse der vorliegenden Arbeit konnten zeigen, dass die Bewertung der korrekten Durchführung der Reanimation in einem Lehrvideo auch für erfahrene NotärztInnen schwierig ist, für Novizen oder gar medizinische Laien kann sie daher kaum möglich sein. Dies ist umso gravierender, da eine präklinische Reanimation vor Eintreffen des Rettungsdiensts in den allermeisten Fällen durch medizinische Laien erfolgt [22]. Gerade für diese Zielgruppe erscheinen somit klar definierte Gütekriterien zur Einordnung der Qualität eines Lehrvideos zur Reanimation notwendig. Studien haben allerdings gezeigt, dass die bisher auf den Plattformen verwendeten Kriterien wie Aufrufe oder Bewertungen durch User nur bedingt als Gütekriterium geeignet sind. So bewerteten beispielsweise Şaşmaz et al. 67 YouTube-Videos zum Erlernen von Traumamanagement anhand von zehn Items, die basierend auf den „ATLS guidelines“ erstellt wurden. Dabei zeigte sich keine Korrelation der Bewertung anhand der Checkliste mit der Anzahl der Aufrufe oder Bewertung auf der Website [18].

Vor diesem Hintergrund ist es wünschenswert, auf Basis einer validierten, auf aktuellen „guidelines“ basierenden Checkliste wie der in der vorliegenden Arbeit erstellten die auf öffentlich zugänglichen Plattformen vorhandenen Videos zu bewerten und diese Bewertung in Form eines Katalogs der empfehlenswerten Videos zu publizieren.

Neben der Bewertung bereits vorhandener Videos kann diese Checkliste aber auch zur Erstellung eigener Videos als Schablone herangezogen werden. Somit wird die korrekte Darstellung der notwendigen Maßnahmen bereits bei der Erstellung des Videos sichergestellt. Die so erstellten Lehrvideos können dann von Studierenden sowohl zur Vorbereitung als auch während einer Lehrveranstaltung genutzt werden. Verschiedene Studien zeigen, dass durch die Nutzung von Lehrvideos das Erlernen praktischer Fertigkeiten verbessert werden kann [9, 11, 13]. Darüber hinaus kann eine Verknüpfung von Gesehenem mit Erlebtem anhand der Videos wiederholt werden [8, 19]. Dies ist besonders für die Reanimation wichtig, da diese in der Realität häufig nicht in einer Situation passiert, die für Studierende adäquat vor- oder nachbereitet werden kann. Somit erscheint in diesem Bereich eine Analyse der Qualität der vorhandenen Videos auf Grundlage einer validierten Checkliste von immenser Bedeutung. Mit der in der vorliegenden Arbeit entwickelten Checkliste wurde erstmals ein Bewertungsinstrument für eine solche Analyse entwickelt.

## Schlussfolgerung

In der vorliegenden Studie konnte erstmalig für den deutschsprachigen Raum eine Checkliste zur Bewertung von Lehrvideos zur Reanimation erstellt und validiert werden. Sie kann nicht nur zur nachträglichen Bewertung bereits vorhandener Videos genutzt werden, sondern gleichzeitig auch bei der Erstellung neuer Videos hilfreich sein.

## Fazit für die Praxis

- Durch die vorliegende Studie existiert nun eine validierte, auf den aktuellen „guidelines“ basierende Checkliste zur Bewertung von Lehrvideos zur Reanimation.
- Die Checkliste kann nicht nur zur nachträglichen Bewertung der Videos dienen, sondern auch bei der Erstellung neuer Videos.
- Die Bewertung der korrekten Durchführung der Maßnahmen der kardiopulmonalen Reanimation in einem Lehrvideo erscheint schon für erfahrene NotärztInnen schwierig. Gerade für diese erscheinen somit klar definierte Gütekriterien zur Einordnung der Qualität eines Lehrvideos zur Reanimation notwendig.
